# Combined effects of Evodiae Fructus point application and psycho-cardiology nursing on post-PCI coronary heart disease patients

**DOI:** 10.3389/fmed.2025.1722004

**Published:** 2025-12-18

**Authors:** Linhua Sun, Li Huang, Chunmei Tong

**Affiliations:** Department of Cardiology, Lujiang County Hospital of TCM, Hefei, China

**Keywords:** Evodiae Fructus point application therapy, psycho-cardiology nursing, coronary heart disease, quality of life, sleeping

## Abstract

**Objective:**

To evaluate the effects of Evodiae Fructus point application combined with psycho-cardiology nursing on sleep, quality of life (QoL), and nursing satisfaction in patients with coronary heart disease after percutaneous coronary intervention (PCI).

**Methods:**

In this randomized controlled trial, 82 patients were allocated to either the study group (*n* = 41), receiving Evodiae Fructus point application and psycho-cardiology nursing alongside conventional care, or the control group (*n* = 41), receiving conventional care alone. The primary outcomes were sleep quality (Pittsburgh Sleep Quality Index, PSQI) and QoL (China Questionnaire of QoL in Cardiovascular patients, CQQC). Secondary outcomes included cardiac function indices (LVEF, LVEDD, LVESD, LVEDV), adverse reactions, and nursing satisfaction.

**Results:**

Cardiac function improved similarly in both groups (e.g., LVEF between-group difference in change: −1.85, 95% CI: −5.42 to 1.72; *p* = 0.305). However, the study group showed superior improvement in the primary endpoints: a lower PSQI total score (between-group difference: −5.56, 95% CI: −7.91 to −3.21; *p* < 0.001) and a higher CQQC total score (between-group difference: 18.45, 95% CI: 10.32 to 26.58; *p* < 0.001). Nursing satisfaction was also higher in the study group (97.56% vs. 90.24%, *p* = 0.023), with no significant difference in adverse reactions (19.5% vs. 9.8%, *p* = 0.322).

**Conclusion:**

For patients undergoing PCI, integrating Evodiae Fructus point application with psycho-cardiology nursing into standard care significantly improves sleep quality, QoL, and nursing satisfaction, with a favorable safety profile. This combined approach did not, however, confer additional short-term benefits to cardiac function.

## Highlights

- This study evaluated the combination of Evodiae Fructus point application and psycho-cardiology nursing in coronary heart disease patients after PCI.
- The intervention significantly improved sleep quality as measured by the Pittsburgh Sleep Quality Index (PSQI).
- Quality of life was notably enhanced in the study group based on CQQC scoring across multiple domains.
- Nursing satisfaction was higher in the combined therapy group compared to conventional care alone.
- The integrative approach proved to be safe, noninvasive, and clinically beneficial for postoperative cardiac rehabilitation.

## Introduction

Coronary heart disease has one of the high incidences of clinical heart diseases. There are many risk factors for coronary heart disease, such as unreasonable diet, lack of exercise, smoking and excessive alcohol consumption. With the continuous improvement of living standards, people in China eat too much animal fat and foods with high cholesterol content, such as animal viscera, all kinds of meat, egg yolk. Emotional excitement and mental stress can also cause the onset of coronary heart disease. The common clinical symptoms of coronary heart disease are chest tightness, angina pectoris, palpitations. With the continuous deterioration and spread of the disease, it is easy to cause serious complications such as heart failure and myocardial infarction (1). At present, percutaneous coronary intervention (PCI) is the main treatment for coronary heart disease, which can alleviate myocardial necrosis and injury and quickly restore myocardial blood perfusion (2).

Evodiae Fructus is a common herbal medicine used in Chinese for thousands of years. It is validated to show promising pharmacological effects on cardiovascular diseases (3). The main activate ingredients from Evodiae Fructus are Dehydroevodiamine, Rutaecarpine, and Evodiamine. Dehydroevodiamine can reduce blood pressure and heart rate in rats (4). Rutaecarpine has a protective effect on cardiac disease by suppressing oxidative stress and apoptosis (5) and can ameliorate cardiac hypertrophy by reducing calcineurin and angiotensin II levels (6). Evodiamine protects the myocardium against atherosclerosis and ischemia–reperfusion induced injury by modulating endothelial-to-mesenchymal transition process (7).

Evodiamine from Evodiae fructus can reduce caffeine-induced sleep disorders in mice in a GABAA-ergic system dependent manner, suggesting its potential role in sleep regulation. Despite these established benefits of PCI and the recognized importance of psychological health, a significant gap remains in the standard perioperative care for these patients. The management of common and distressing symptoms like insomnia often relies on pharmacological agents, which can carry risks of side effects and dependence. Non-pharmacological, complementary approaches are needed. Psycho-cardiology model is an important medical field that studies the correlation between cardiovascular diseases and mental disorders. Rehabilitation of patients with cardiovascular diseases can be effectively promoted by adjusting their mental state (8). While the psycho-cardiology model is gaining recognition, its integration into structured, routine nursing care for PCI patients is not yet widespread. The potential synergistic effects of combining a simple, non-invasive Traditional Chinese Medicine (TCM) modality like Evodiae Fructus point application with a systematic psycho-cardiology nursing protocol have been underexplored.

Therefore, this study aimed to bridge this gap by conducting a randomized controlled trial to evaluate the clinical effects of integrating Evodiae Fructus point application with psycho-cardiology nursing into conventional care. We hypothesized that this combined intervention would be more effective than conventional care alone in improving sleep quality and quality of life—key patient-centered outcomes—and in enhancing nursing satisfaction for patients undergoing PCI during the perioperative period, without increasing the burden of adverse reactions.

## Methods

### Study design and ethics

This was a prospective, randomized, assessor-blinded, single-center clinical trial conducted at the Department of Cardiology, Lujiang County Hospital of TCM. The study was approved by the Ethics Committee of Lujiang County Hospital of Traditional Chinese Medicine (Approval No.: 2020–106; Date: October 22, 2020) and was registered at the Chinese Clinical Trial Registry. Written informed consent was obtained from all individual participants prior to enrollment. All procedures performed in this study were conducted in accordance with the principles of the Declaration of Helsinki.

### Participants and randomization

A total of 82 patients with coronary heart disease scheduled for PCI between January 2021 and October 2023 were recruited. Participants were randomly assigned in a 1:1 ratio to either the study group or the control group using a computer-generated random number sequence. The allocation sequence was concealed using sequentially numbered, opaque, sealed envelopes (SNOSE), which were opened only after the participant had provided consent and completed baseline assessments. The outcome assessors (e.g., sonographers for echocardiography) were blinded to group allocation.

Inclusion criteria: (1) Diagnosed with coronary heart disease according to the ESC 2019 guidelines for the diagnosis and management of chronic coronary syndromes (9); (2) Age > 20 years old; (3) Scheduled for elective PCI; (4) Provided written informed consent.

Exclusion criteria: (1) Patients with severe cardiac mechanical complications (e.g., ventricular septal rupture, severe mitral regurgitation); (2) Patients with blood system disorders and severe autoimmune diseases; (3) Patients with hepatic or renal insufficiency (e.g., ALT/AST > 3 times upper limit of normal, eGFR < 30 mL/min/1.73m^2^); (4) Evidence of active clinical infection or fever; (5) Chronic inflammatory conditions or unhealed wounds, especially on the feet; (6) History of major surgery within the past 3 months; (7) Known allergy to Evodiae Fructus or plaster components.

### Interventions

Control group: Patients in the control group received conventional pharmacotherapy and routine nursing care according to the hospital’s standard PCI protocol. The drug regimen included: Enteric-coated aspirin tablets (100 mg, once daily); Atorvastatin calcium tablets (10 mg, once daily); Isosorbide mononitrate capsules (40 mg, once daily); Metoprolol tartrate tablets (12.5 mg, twice daily); for patients with poor cardiac function, digoxin (0.25 mg, once daily) was added. Routine nursing included health education, medication guidance, diet and exercise advice, and ward environment management.

Study group: In addition to the identical conventional care received by the control group, patients in the study group received the combined intervention of Evodiae Fructus point application and structured psycho-cardiology nursing for one week.

Evodiae Fructus Point Application: (1) Preparation: Dried Evodiae Fructus (voucher specimen deposited in our TCM pharmacy, Batch No.: 0324) was ground into a fine powder (80-mesh sieve). For each application, 5 g of the powder was thoroughly mixed with white vinegar (Zhenjiang brand, 5% acidity) to form a homogeneous paste. (2) Application: The paste was applied to the center of a specialized, hypoallergenic acupoint plaster (6 × 7 cm, Registration No.: 20180023), covering an area of 3 × 3 cm with a thickness of approximately 1.5 cm. Patients were instructed to clean the soles of their feet before application. The plaster was applied bilaterally to the Yongquan acupoint (KI1), located at the anterior part of the sole in the depression when the foot is curled, approximately at the junction of the anterior one-third and posterior two-thirds of the line connecting the base of the 2nd and 3rd toes and the heel. The application was performed once daily for 4–6 h, with a total course of 7 days.

Structured psycho-cardiology nursing protocol:

A standardized yet individualized protocol was delivered by trained nurses and included the following core components: (1) Initial Psychological Assessment and Active Communication (20–30 min on day 1): Conducted in a quiet, private room. Nurses used empathetic communication and active listening to understand patient concerns. Guided relaxation techniques (e.g., controlled breathing, pleasant imagery) were taught. (2) Environmental Optimization and Demystification (Ongoing): The ward environment (temperature, humidity, noise, light) was optimized. The purpose and sounds of monitoring/ rescue equipment were explained in detail to reduce fear. (3) Individualized Psychological Intervention (15–20 min, at least twice weekly): For patients with significant anxiety or depression (identified via initial assessment or clinical observation), targeted counseling was provided. This involved cognitive restructuring, positive reinforcement, and careful listening to build rapport and improve compliance. (4) Structured Health Education (30–45 min, once as a group session): A structured lecture using presentations and demonstrations covered CHD pathophysiology, PCI procedure, medication importance, risk factor management, and rehabilitation goals. A Q&A session encouraged discussion and peer support.

### Outcomes and measures

Primary Outcomes: (1) Sleep Quality: Assessed using the Pittsburgh Sleep Quality Index (PSQI) (10). The total score (range 0–21, higher scores indicating worse quality) was the primary sleep metric. (2) Quality of Life (QoL): Assessed using the China Questionnaire of Quality of Life in Patients with Cardiovascular Diseases (CQQC) (11). The total score (range 0–161, higher scores indicating better QoL) was the primary QoL metric.

Secondary Outcomes: (1) Cardiac Function: Measured using a Resona 7 Mindray Color Doppler Ultrasound System by a sonographer blinded to group allocation. Parameters included Left Ventricular Ejection Fraction (LVEF), Left Ventricular End-Systolic Diameter (LVESD), Left Ventricular End-Diastolic Diameter (LVEDD), and Left Ventricular End-Diastolic Volume (LVEDV). (2) Nursing Satisfaction: Evaluated on the last day of the intervention using a self-developed satisfaction survey (100-point scale) covering nursing attitude, effectiveness, health guidance, and psychological support. Scores ≥80 were “Very Satisfied,” 60–79 were “Generally Satisfied,” and <60 were “Unsatisfied.” The total satisfaction rate was calculated. (3) Adverse Reactions: Monitored and recorded throughout the study period, including nausea, vomiting, drowsiness, deep vein thrombosis, and local skin reactions at the application site (e.g., redness, itching).

All outcome assessments (PSQI, CQQC, satisfaction survey) were conducted at baseline (before PCI) and one month after the nursing intervention. Cardiac function was measured at baseline and within one week after the completion of the intervention.

### Statistical methods

Data analysis was performed using SPSS version 28.0. The sample size was determined based on feasibility for this single-center pilot study; a formal power calculation was not conducted. Continuous data were presented as mean ± standard deviation (SD) and analyzed for between-group differences using independent-sample t-tests (for post-intervention scores) or Analysis of Covariance (ANCOVA) with baseline scores as a covariate, which is more powerful. Within-group differences were analyzed using paired t-tests. Categorical data were expressed as frequencies and percentages and compared using the Chi-square test or Fisher’s exact test, as appropriate. The primary analysis was the intention-to-treat (ITT) analysis. No imputation was performed for missing data as complete datasets were obtained. A two-sided *p*-value < 0.05 was considered statistically significant. Given the multiple comparisons for CQQC subdomains and PSQI components, a Bonferroni correction was applied for these specific analyses, setting the significance level at *p* < 0.005.

## Results

### Baseline characteristics and patient flow

During the study period, 94 patients were assessed for eligibility. Twelve patients were excluded (8 did not meet inclusion criteria, 4 declined to participate), resulting in 82 patients who were randomized. All 82 patients completed the trial and were included in the final intention-to-treat analysis, with no loss to follow-up or missing data. The baseline demographic and clinical characteristics were well-balanced between the two groups, with no statistically significant differences observed (all *p* > 0.05), as detailed in Supplementary Table S1.

### Comparison of cardiac functions in two groups

As shown in Table 1, both groups showed significant improvements in all cardiac function parameters from baseline to post-treatment (all *p* < 0.01). However, there were no statistically significant differences in the degree of improvement between the study group and the control group for LVEF (Between-group difference in change: -1.85, 95% CI: −5.42 to 1.72; *p* = 0.305), LVESD, LVEDD, or LVEDV.

**Table 1:** Comparison of cardiac function parameters (mean ± SD).

Group	Time point	LVEF (%)	LVESD (mm)	LVEDD (mm)	LVEDV (mL)
Control	Before	33.34 ± 4.78	39.16 ± 10.12	47.18 ± 7.93	263.17 ± 17.36
(*n* = 41)	After	50.13 ± 8.16*	28.84 ± 4.42*	36.58 ± 6.27*	210.35 ± 14.29*
Study	Before	34.47 ± 5.02	40.28 ± 9.41	46.43 ± 8.11	258.82 ± 19.42
(*n* = 41)	After	48.28 ± 9.27*	27.69 ± 4.83*	37.15 ± 6.94*	213.25 ± 14.11*
Between-group difference in change (95% CI)		-1.85 (−5.42 to 1.72)	−0.92 (−3.45 to 1.61)	1.02 (−1.85 to 3.89)	4.15 (−2.89 to 11.19)
*p*-value (between-group)		0.305	0.472	0.481	0.245

*p < 0.01 compared to before treatment within the same group.

### Comparison of sleeping conditions of two groups

The study group demonstrated significantly greater improvement in sleep quality compared to the control group. The post-intervention PSQI total score was markedly lower in the study group (9.11 ± 2.84 vs. 14.67 ± 6.52; between-group difference: -5.56, 95% CI: −7.91 to −3.21; *p* < 0.001). Significant between-group differences were also observed across all PSQI components after Bonferroni correction (*p* < 0.005), as detailed in Table 2.

**Table 2:** Comparison of post-intervention PSQI scores (mean ± SD).

PSQI component	Control group (*n* = 41)	Study group (*n* = 41)	Between-group difference (95% CI)	*p*-value
Sleep quality	2.44 ± 1.12	1.35 ± 0.84	−1.09 (−1.52 to −0.66)	<0.001*
Sleep latency	1.97 ± 0.91	1.07 ± 0.61	−0.90 (−1.25 to −0.55)	<0.001*
Sleep duration	2.12 ± 1.21	1.45 ± 0.91	−0.67 (−1.13 to −0.21)	0.005*
Sleep efficiency	2.27 ± 0.89	1.12 ± 0.66	−1.15 (−1.50 to −0.80)	<0.001*
Sleep disturbance	2.18 ± 0.82	1.45 ± 0.45	−0.73 (−1.00 to −0.46)	<0.001*
Use of sleep medication	1.79 ± 0.88	1.12 ± 0.85	−0.67 (−1.04 to −0.30)	0.001*
Daytime dysfunction	1.88 ± 0.75	1.53 ± 0.78	−0.35 (−0.68 to −0.02)	0.038
PSQI total score	14.67 ± 6.52	9.11 ± 2.84	−5.56 (−7.91 to −3.21)	<0.001*

Statistically significant after Bonferroni correction for multiple comparisons (significance level set at *p* < 0.005).

### Comparison of quality-of-life of two groups

Post-intervention CQQC scores were significantly higher in the study group across all domains after Bonferroni correction (p < 0.005), indicating a superior improvement in QoL. The between-group difference in the total CQQC score was 18.45 points (95% CI: 10.32 to 26.58; *p* < 0.001). Detailed results are presented in Table 3.

**Table 3:** Comparison of post-intervention CQQC scores (mean ± SD).

CQQC domain	Control group (*n* = 41)	Study group (*n* = 41)	Between-group difference (95% CI)	*p*-value
Physical condition	10.12 ± 3.02	12.67 ± 3.14	2.55 (1.21 to 3.89)	<0.001*
Treatment status	11.14 ± 1.87	12.36 ± 1.96	1.22 (0.41 to 2.03)	0.004*
Social psychology	38.16 ± 4.95	42.29 ± 4.82	4.13 (2.01 to 6.25)	<0.001*
Disease situation	27.62 ± 3.85	30.56 ± 4.08	2.94 (1.21 to 4.67)	0.001*
Daily life	28.27 ± 2.63	31.15 ± 3.09	2.88 (1.67 to 4.09)	<0.001*
Work condition	9.73 ± 1.23	11.36 ± 1.18	1.63 (1.12 to 2.14)	<0.001*
CQQC total score	125.04 ± 12.51	140.39 ± 14.27	15.35 (9.45 to 21.25)	<0.001*

Statistically significant after Bonferroni correction for multiple comparisons (significance level set at *p* < 0.005).

### Comparison of adverse reactions of two groups

The overall incidence of adverse reactions was not significantly different between the study group and the control group (19.5% vs. 9.8%, *p* = 0.322). In the study group, three patients (7.3%) reported mild, transient skin redness at the application site, which resolved spontaneously without discontinuing treatment. Other systemic adverse events were infrequent and comparable between groups, as detailed in Table 4.

**Table 4:** Incidence of adverse reactions [n (%)].

Adverse reaction	Control group (*n* = 41)	Study group (*n* = 41)	*p*-value
Nausea	2 (4.9%)	3 (7.3%)	1.000
Vomiting	0 (0%)	1 (2.4%)	1.000
Drowsiness	1 (2.4%)	3 (7.3%)	0.617
Deep vein thrombosis	1 (2.4%)	1 (2.4%)	1.000
Local skin reaction	0 (0%)	3 (7.3%)	0.241
Total patients with ≥1 AE	4 (9.8%)	8 (19.5%)	0.322

*p*-values calculated using Fisher’s exact test due to small expected cell counts.

### Comparison of nursing satisfaction of two groups

The overall nursing satisfaction rate was significantly higher in the study group (97.56%) than in the control group (90.24%), with a between-group difference of 7.32% (95% CI: 0.92 to 13.72%; *p* = 0.023). The distribution of satisfaction levels is shown in Table 5.

**Table 5:** Comparison of nursing satisfaction [*n* (%)].

Satisfaction level	Control group (*n* = 41)	Study group (*n* = 41)
Very satisfied	17 (41.5%)	29 (70.7%)
Generally satisfied	20 (48.8%)	11 (26.8%)
Unsatisfied	4 (9.8%)	1 (2.4%)
Total satisfaction rate	37 (90.24%)	40 (97.56%)
Between-group difference (95% CI)	7.32% (0.92 to 13.72%)	
*p*-value	0.023	

## Discussion

This randomized controlled trial demonstrated that integrating Evodiae Fructus point application with a structured psycho-cardiology nursing protocol into conventional post-PCI care led to significant and clinically meaningful improvements in patient-centered outcomes. Specifically, the combined intervention resulted in superior sleep quality, enhanced overall quality of life, and higher nursing satisfaction compared to conventional care alone. These benefits were achieved without a significant increase in adverse reactions. Notably, the intervention did not confer additional short-term improvements in cardiac function parameters beyond those achieved with standard medical therapy.

The pronounced improvement in PSQI scores aligns with the traditional use of Evodiae Fructus (Wu Zhu Yu) for calming the spirit and its documented application for insomnia. The Yongquan acupoint (KI1), to which the herb was applied, is a well-established point in TCM for guiding Qi downward and calming the mind (12, 13). Our findings provide clinical evidence supporting this traditional practice. Furthermore, preclinical studies have suggested that active components like evodiamine may modulate sleep architecture through the GABAA system (14). While our study was not designed to elucidate the underlying mechanism, the significant sleep improvement observed provides clinical support for further investigation into these potential pathways.

The significant enhancement in CQQC scores across all domains underscores the value of a holistic, biopsychosocial approach. The psycho-cardiology nursing component directly addressed the psychological distress commonly experienced by PCI patients, which is a known predictor of poorer QoL (8, 15). By providing empathetic communication, psychological support, and comprehensive health education, this model likely empowered patients, reduced fear, and improved coping strategies, thereby enhancing their perceived physical state, social function, and daily living activities. The synergy between the physiological calming effect hypothesized from the acupoint application and the psychological support from the nursing care likely contributed to this robust QoL improvement.

A pivotal finding of our study was the lack of a significant between-group difference in cardiac function indices. This is a crucial result that warrants careful interpretation. It suggests that the benefits of the combined intervention are primarily manifested in patient-reported outcomes (PROs) like sleep and QoL, rather than in reverse remodeling of the heart in the immediate post-PCI period. This is biologically plausible for several reasons. First, the powerful effects of evidence-based pharmacotherapy (e.g., beta-blockers, statins, ACEIs) on cardiac function are well-established and likely constituted the primary driver of myocardial recovery in both groups, potentially overshadowing any incremental effect from our non-pharmacological intervention within a short timeframe (2). Second, the pathways through which our intervention worked—primarily sleep regulation and psychological distress reduction—may not directly translate into improved LVEF or ventricular dimensions over a one-month period. This dissociation between PROs and “hard” physiological parameters is recognized in cardiovascular research and highlights that these interventions target different aspects of the patient’s health experience (16, 17).

Our results regarding sleep and QoL improvements are consistent with a growing body of evidence supporting integrative therapies in cardiac rehabilitation. For instance, a study by Zhen L. (2024) also found that a psycho-cardiology model significantly improved psychological well-being in post-PCI patients (18). Similarly, the benefits of acupoint stimulation for symptom management in CHD patients have been reported (12, 13). However, our study extends the existing literature by systematically evaluating the combination of a specific TCM modality with a structured psycho-cardiology protocol, demonstrating a synergistic effect on key PROs. This approach aligns with the growing call for integrated, multimodal non-pharmacological strategies in comprehensive cardiac care (19). The high nursing satisfaction rate in our study group further reinforces the feasibility and acceptability of integrating such complementary approaches into standard nursing workflows, a finding echoed in studies of patient-centered care models (11).

Notwithstanding the positive findings, this study has several limitations that should be considered when interpreting the results. (1) Lack of patient and staff blinding: Due to the nature of the behavioral and point application interventions, it was not feasible to blind patients or nursing staff to group allocation. This introduces a potential for performance and detection bias, particularly for subjective patient-reported endpoints like the PSQI and CQQC, although objective outcome assessors (e.g., for cardiac ultrasound) were blinded. (2) Sample size and single-center design: The sample size was modest and based on feasibility rather than a formal power calculation. Conducted at a single center, the findings may lack generalizability to other populations or healthcare settings. (3) Short follow-up period: The one-month follow-up period for PROs is relatively short. It remains unknown whether the observed benefits in sleep and QoL are sustained in the long term. (4) Intervention standardization: While a structured protocol was used, the psycho-cardiology nursing involved individualized interactions, which, while clinically valuable, introduces an element of variability that is difficult to fully standardize. (5) Exploration of mechanisms: This study was designed as an efficacy trial and did not include measures (e.g., plasma catecholamines, inflammatory markers) to explore the biological mechanisms underlying the observed clinical benefits.

The findings suggest that the combined protocol is a safe, non-pharmacological, and effective adjunct to conventional care for improving the subjective well-being of post-PCI patients. Future research should focus on confirming these results in larger, multi-center trials with longer follow-up periods. Studies incorporating sham acupoint controls would help isolate the specific effect of the Evodiae Fructus application. Furthermore, research exploring the biochemical and physiological pathways (e.g., stress hormones, autonomic nervous system balance, inflammatory markers) through which these interventions exert their effects would be highly valuable.

## Conclusion

In this study, the integration of Evodiae Fructus point application therapy with a structured psycho-cardiology nursing protocol into conventional care demonstrated significant benefits for key patient-centered outcomes within the one-month study period. These findings suggest that this non-pharmacological, integrative approach is a valuable and feasible adjunct to standard post-PCI management for enhancing subjective patient well-being in the perioperative period. Future studies are warranted to confirm these results in larger, longer-term trials and to explore the mechanisms of action.

## Data Availability

The datasets presented in this study can be found in online repositories. The names of the repository/repositories and accession number(s) can be found in the article/Supplementary material.
