# Supplementary material for: Combined effects of Evodiae Fructus point application and psycho-cardiology nursing on post-PCI coronary heart disease patients
Source: Front Med (Lausanne). 2025 Dec 18;12:1722004. doi: 10.3389/fmed.2025.1722004 (PMC12756173; doi:10.3389/fmed.2025.1722004)
Supplement: Supplementary file 1 [file Table_1.docx]

**Table S1 Baseline demographic and clinical characteristics of the participants**

| **Characteristic** | **Control Group (n=41)** | **Study Group (n=41)** | **p-value** |
| --- | --- | --- | --- |
| Age, years (mean ± SD) | 67.46 ± 9.38 | 63.73 ± 8.25 | 0.054 |
| Gender, Male (n, %) | 25 (61.0%) | 26 (63.4%) | 0.818 |
| Length of Stay, days (mean ± SD) | 10.10 ± 2.24 | 8.46 ± 1.45 | <0.001* |
| Comorbidities (n, %) |  |  |  |
| - Hypertension | 28 (68.3%) | 25 (61.0%) | 0.485 |
| - Diabetes Mellitus | 15 (36.6%) | 12 (29.3%) | 0.481 |
| - Hyperlipidemia | 22 (53.7%) | 24 (58.5%) | 0.657 |
| Baseline LVEF, % (mean ± SD) | 33.34 ± 4.78 | 34.47 ± 5.02 | 0.290 |
| Baseline PSQI Total Score (mean ± SD) | 14.25 ± 3.81 | 13.89 ± 3.45 | 0.649 |
| Baseline CQQC Total Score (mean ± SD) | 89.11 ± 12.35 | 91.80 ± 13.62 | 0.351 |

Note: The significant difference in length of stay is expected as it is an outcome of the intervention, not a baseline characteristic. It is included here for descriptive completeness but was not tested for balance.
